# A Hospital Chairman's Grievances

**Published:** 1912-02-24

**Authors:** 


### A Hospital Chairman's Grievances.

### I. COMPLAINTS AND THEIR INVESTIGATION.

How is it, I wonder that so many people delight to get a complaint against a hospital? The preponderance of thanks numerically over complaints on the part of patients is enormous. Let anyone go round a well-conducted voluntary hospital and get into quiet chat with the patients, either in the beds or with the more convalescent sitting round the fire, never will they hear a jarring note! Gratitude everywhere!

Yet if by chance an inquest be held on a patient whom, for some reason or other the physicians and surgeons have not seen fit to admit, or who may have expired after discharge, the neighbourhood is aroused; and one would think folks were talking about atrocities too bad to be named. They attack the Lay Committee, sending blue pencilled cuttings from the local Press marked "disgraceful," "disgusting," and worse. Oft times, too, the Lay Committee condemn their own Institution without benefit of trial or inquiry.

The fact is too little appreciated, even by Lay Governors themselves, that they have no legal responsibility on matters connected with treatment. Their responsibility ends with the appointment of proper responsible and duly qualified officers, voluntary or resident. Therefore, even should mistakes have been made—a not improbable supposition considering the large numbers treated and the complications of the human organism—they have no actual right to acquit or to condemn. Of course, if scandalous misbehaviour or ineptness were disclosed on the part of any medical officer, the Lay Committee would have every reason to ask him to resign and would do so.

As a chairman, it has long been a question with me, whether or no to investigate complaints. I have, however, always (when possible) done so personally and in conjunction with a member of the honorary staff: and we have had some funny experiences. The total result of such investigations has developed the greatest respect for the modern young resident physician or surgeon.

I remember when the medical student, just qualified and appointed house surgeon, frequently had a cock-sureness and thought himself as good as the best, and sometimes liked to show the patients what small beer he considered them. He used sometimes, too, to be a man to whom strong language was so natural that he seldom called a spade a spade. All this has changed: the modern "house man" has, as a rule, a quiet and dignified sense of responsibility and his attention to detail is generally most commendable. We have often been able to refute a most ingeniously-pieced-together grievance against the hospital, and have been helped so to do by unanswerable evidence produced with courtesy and frankness from a house man; but I never pose as having any right to any information as to facts regarding treatment, merely asking them to help if they feel inclined, as complaints make the getting of money so difficult; this approach is invariably met more than half way.

What vexes one more than anything else is the attitude that coroners, sometimes, and their juries nearly always, assume. Frequently statements are allowed to go unchallenged from the jury to the Press, unfounded on one iota of legal evidence; then all the neighbourhood says, "disgraceful," "disgusting," and the majority fails to see or to notice any disclaimer that the hospital is able to achieve. This, in spite of the fact that a modern coroner is supposed to have a medico-judicial education. However, "il faut souffrir pour être belle," and I suppose the beauty of self-sacrifice is one of the most blessed rewards of the voluntary worker. Still, one grudges the penny or halfpenny stamp on the anonymous condemnatory effusion. Good could be done with the coin if slipped into the hospital box.

Chairman.

